# Implementation of an international standardized set of outcome indicators in pregnancy and childbirth in Kenya: Utilizing mobile technology to collect patient-reported outcomes

**DOI:** 10.1371/journal.pone.0222978

**Published:** 2019-10-16

**Authors:** Ishtar Al-Shammari, Lina Roa, Rachel R. Yorlets, Christina Akerman, Annelies Dekker, Thomas Kelley, Ramona Koech, Judy Mutuku, Robert Nyarango, Doriane Nzorubara, Nicole Spieker, Manasi Vaidya, John G. Meara, David Ljungman

**Affiliations:** 1 International Consortium for Health Outcomes Measurement (ICHOM), Boston, Massachusetts, United States of America; 2 Program in Global Surgery and Social Change, Harvard Medical School, Boston, Massachusetts, United States of America; 3 Department of Obstetrics & Gynecology, University of Alberta, Edmonton, Canada; 4 Department of Plastic & Oral Surgery, Boston Children’s Hospital, Boston, Massachusetts, United States of America; 5 PharmAccess (PAI), Amsterdam, The Netherlands; 6 Gertrude’s Children’s Hospital, Nairobi, Kenya; 7 Department of Surgery, the Sahlgrenska Academy at University of Gothenburg, Gothenburg, Sweden; Bielefeld University, GERMANY

## Abstract

**Background:**

Limited data exist on health outcomes during pregnancy and childbirth in low- and middle-income countries. This is a pilot of an innovative data collection tool using mobile technology to collect patient-reported outcome measures (PROMs) selected from the International Consortium of Health Outcomes Measurement (ICHOM) Pregnancy and Childbirth Standard Set in Nairobi, Kenya.

**Methods:**

Pregnant women in the third trimester were recruited at three primary care facilities in Nairobi and followed prospectively throughout delivery and until six weeks postpartum. PROMs were collected via mobile surveys at three antenatal and two postnatal time points. Outcomes included incontinence, dyspareunia, mental health, breastfeeding and satisfaction with care. Hospitals reported morbidity and mortality. Descriptive statistics on maternal and child outcomes, survey completion and follow-up rates were calculated.

**Results:**

In six months, 204 women were recruited: 50% of women returned for a second ante-natal care visit, 50% delivered at referral hospitals and 51% completed the postnatal visit. The completion rates for the five PROM surveys were highest at the first antenatal care visit (92%) and lowest in the postnatal care visit (38%). Data on depression, dyspareunia, fecal and urinary incontinence were successfully collected during the antenatal and postnatal period. At six weeks postpartum, 86% of women breastfeed exclusively. Most women that completed the survey were very satisfied with antenatal care (66%), delivery care (51%), and post-natal care (60%).

**Conclusion:**

We have demonstrated that it is feasible to use mobile technology to follow women throughout pregnancy, track their attendance to pre-natal and post-natal care visits and obtain data on PROM. This study demonstrates the potential of mobile technology to collect PROM in a low-resource setting. The data provide insight into the quality of maternal care services provided and will be used to identify and address gaps in access and provision of high quality care to pregnant women.

## Introduction

Reducing maternal and neonatal mortality are global health priorities set by Sustainable Development Goals (SDG) target 3.1 and 3.2 [1]. It is also essential to decrease morbidity as 10–20 million women develop disabilities every year from complications of pregnancy or poor quality of care[2–4]. Poor pregnancy and childbirth outcomes disproportionately affect women living in low- and middle-income countries (LMICs)[5, 6], where high-quality facilities are few and difficult to reach due to financial and social barriers [7, 8]. As efforts to increase access continue, it is crucial to focus on measuring and improving the quality of services provided to women and neonates[9, 10].

Quality of care is multi-faceted, making it difficult to measure accurately[11]. In LMICs, difficulties in measuring quality are compounded by lack of long-term patient follow-up. The Donabedian quality framework has been widely used to capture the breadth of quality healthcare in the dimensions of structure, process, and outcomes[12]. In LMICs, quality during pregnancy and childbirth has focused mostly on the *Structure* and *Process* dimensions. However, these do not guarantee quality of care provided[13, 14]. The *Outcome* dimension has focused on maternal mortality and maternal near-miss cases [15, 16]. In addition to mortality and severe morbidity, quality of life throughout pregnancy and childbirth is essential, but few efforts have been made to measure patient-reported outcome measures (PROM) related to depression, incontinence, breastfeeding, dyspareunia[6], and patient satisfaction in LMICs [6, 17, 18]. Some indicators of quality obstetric care have been measured retrospectively via household surveys[19–21], but prospective data collection during pregnancy and childbirth is lacking. A consensus on measurement of non-severe maternal outcomes is essential to make comparisons at the national and international level and to effectively allocate limited resources for quality improvement.

Value-based healthcare (VBHC) advocates for patient-centered care and defines value as health outcomes achieved per dollar spent[22]. Transparency of the outcomes achieved promotes dialogue around the variations in outcomes and learning from best practices, to optimize quality and value for patients. The International Consortium for Health Outcomes Measurement (ICHOM) translates the theory of VBHC into practice by defining global Standard Sets of outcomes, such as for Pregnancy and Childbirth (PCB), that aim to reflect what matters most to patients[23]. To our knowledge implementation of this Standard Set had not been applied in a marginalized population in LMIC.

Kenya has a high burden of maternal and neonatal mortality. Estimates from 2015 revealed a MMR of 510 deaths per 100,000 live births and infant mortality of 37 per 1000 live births[24]. In order to meet the SDG targets, it will have to decrease these to 70 maternal deaths per 100,000 births and 12 neonatal deaths per 1000 births[25]. In 2013, the government introduced a defined set of free maternity services at all levels of care in the public health sector[26]. Despite increases in skilled birth attendance and antenatal care, maternal and neonatal mortality remains high, likely reflective of the quality of services provided [27, 28]. Most Kenyan women deliver at public health facilities, and quality of maternal care has been found to be lower for impoverished women[28]. Attempts at improving quality, such as the Ministry of Health’s Kenya Quality Model for Health, have been limited by a slow uptake and focus on availability of resources instead of utilization or quality of service provision[21]. Effective coverage, which measures the performance of health systems[29], of maternal and child services remains low in Kenya despite increases from 26.7% to 50.9% from 2003 to 2014, leaving half of the women without effective coverage[21].

In 2011, approximately 93% of Kenyans used a mobile phone and 67% were mobile money customers[30]. In the same year, the government launched the Kenya National eHealth Strategy 2011–2017, which highlights five specific strategic areas of focus, including improving access to health through the use of mobile devices[31]. Mobile health (mHealth) is a rapidly-growing sector that aims to center care on the patient by overcoming barriers to access and empowering patients to play an active role in their health[32]. In Kenya, mHealth has been successfully used to improve adherence to medication and patient involvement in care[33, 34].

Drawing on the rise of mHealth and the urgent need to measure and improve quality of maternity care, we piloted an innovative data capture technique for the collection of the ICHOM’s Pregnancy and Childbirth Standard Set across informal settlements in Nairobi. Our objectives are to adapt the ICHOM Pregnancy and Childbirth Standard for use in Nairobi’s informal settlements, to determine the feasibility of using a mobile phone platform to follow up patients and collect patient-reported outcome measures (PROM) in a population with otherwise limited access to longitudinal care.

## Methods

### Study design

This is a pilot study on the feasibility of implementation of the ICHOM Pregnancy and Childbirth (PCB) Standard Set in a prospective cohort of mothers in Nairobi, Kenya using mHealth technology. Focus groups were conducted to adapt the ICHOM PCB Standard Set to the local setting. A mobile wallet M-TIBA and a mobile platform mSurvey were adapted and integrated to collect administrative and PROM data. Administrative and PROM data were collected prospectively.

### Adaptation of ICHOM PCB Standard Set to the local setting

**Fig 1:**
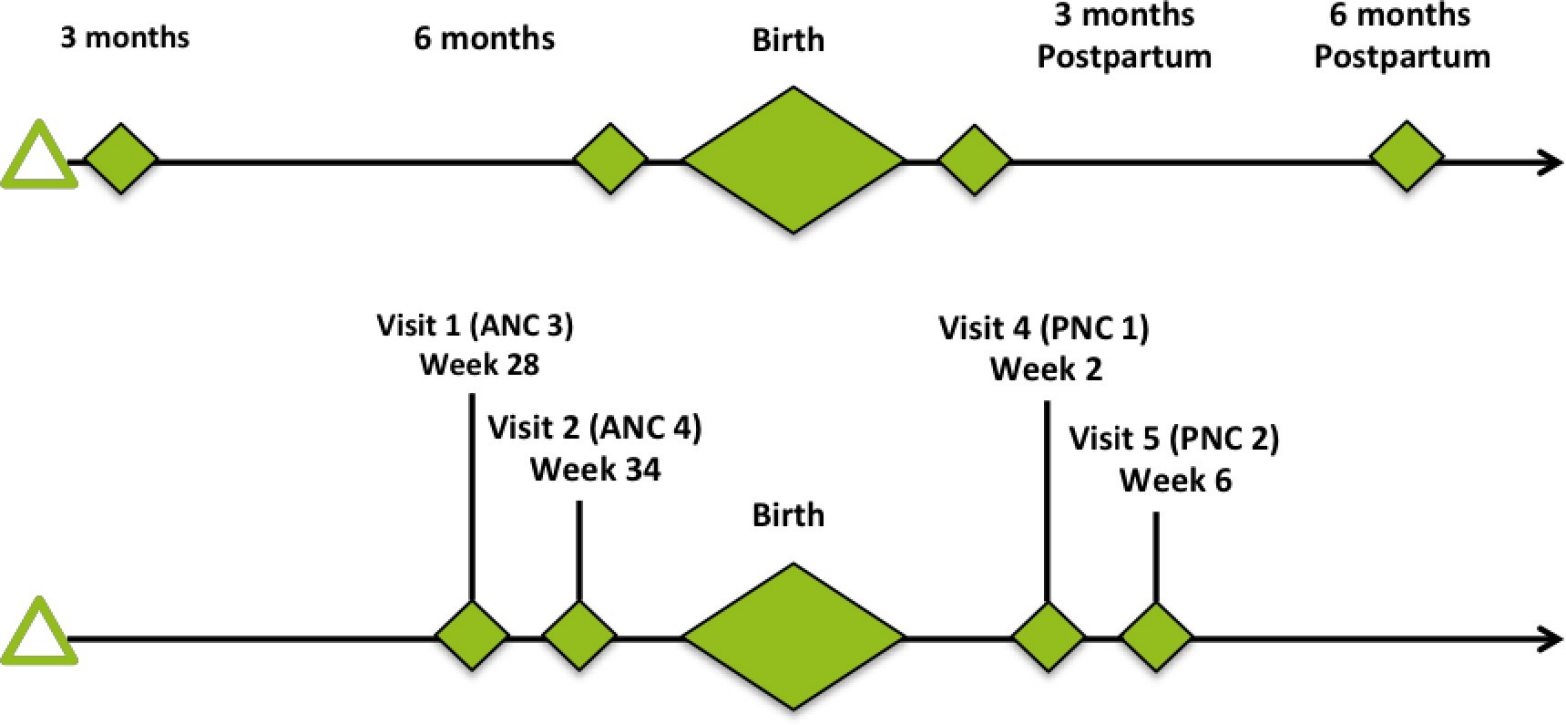
Adaptation of pilot timeline. The top timeline is recommended by the ICHOM Pregnancy and Childbirth Standard Set and spans from the third month of pregnancy until 6 months postpartum. The revised timeline adapted to the Kenyan context and used in the pilot starts at the 28th week of pregnancy and ends 6 weeks postpartum. The new timeline was adopted in line with the existing patient pathway for maternal health management in Nairobi. The green diamonds represent data collection points that coincide with clinic visits.

The ICHOM PCB Standard Set collects data from women during pregnancy, delivery, and up to six weeks postpartum, contains hospital-reported data, seven domains within patient-reported outcomes, and a set of case-mix factors[23]. A committee of medical experts and stakeholders reviewed the indicators for implementation based on level of importance, social and cultural acceptability. In the first-round medical research experts from Kenya, Ghana and the Netherlands conducted a feasibility assessment and a preliminary selection of outcome indicators to pilot; considerations included literacy, language spoken, and constraints of using a mobile platform. The selected indicators were presented and finalized in the second round at a workshop in Nairobi to key stakeholders from the private and public Kenyan healthcare sector. When required, licensing was requested for the selected PROM tools (ICIQ-SF, Wexner, PROMIS SFFAC102 and PHQ-2). The timeline for data collection was modified from the original ICHOM timeline which recommends the first survey to be administered during the first trimester, since this is a proof of concept pilot we adapted the timeline to recruit women in the third trimester (Fig 1).

### Mobile health tools

The M-TIBA mobile wallet was developed by PharmAccess Group in partnership with Safaricom and CarePay as a virtual wallet for healthcare expenses. It contains funds that can only be used for healthcare by selected providers across Kenya. The connected health care providers are all enrolled in the SafeCare program which comprises healthcare standards accredited by the International Society for Quality in Health Care (ISQua) for healthcare providers in LMICs. The M-TIBA platform collects real-time medical and financial data on health care transactions and allows for measurement of administrative outcome indicators.

mSurvey is a Nairobi-based start-up that collects consumer data from communities that are difficult to reach; given this capability, it was utilized to collect PROM from women in Nairobi settlements through the mobile phone platform. M-TIBA allowed participants to access healthcare financing thus reducing the financing barriers to care. For this pilot project, M-TIBA and mSurvey were connected through an application-programming interface to link PROMs to administrative data and financial data while ensuring privacy and confidentiality of patients. At each facility, the M-TIBA account is opened upon patient registration, and upon completion of the visit, the closing of the M-TIBA account triggers the delivery of the mSurvey to patients, therefore linking clinical and administrative data from M-TIBA with the ICHOM Set on the mSurvey. Participants had 5 days to complete the questionnaire before receiving an automated reminder. Once the mSurvey was developed, a focus group consisting of 12 women who met inclusion criteria was conducted to test its use as a tool for PROM data collection. Focus group participants received 10 outcome questions via text on their mobile phones in order to test the mobile platform. They were each given 20 minutes to respond to the survey. After which, participants were asked about their experience utilizing the mobile platform. Participants provided feedback that fell into five main themes including: (1) English was a barrier for some participants, (2) for most this was their first experience with mobile surveys, (3) concerns for desirability bias, (4) willingness to participate especially if responses were integrated into clinical care and (5) positive feedback on potential for benefiting from survey with added education and awareness around pregnancy and childbirth.

As a result, the need for initial support using the mobile application was identified and patient liaison officers (PLO) were recruited for the enrollment process. PLOs also followed up women who did not present to clinic to reduce bias from loss to follow up.

### Setting

Participating facilities that provide ANC, PNC and/or delivery services were chosen based on their previous enrollment as M-TIBA facilities and participants of PharmAccess’ SafeCare quality improvement program. In total, four pilot facilities were selected; two outreach clinics from Gertrude’s Children’s Hospital (Githogoro and Mathare clinics), and two referral hospitals, Jacaranda Maternity and Nazareth Hospital. The clinics provide approximately 2,000 ANC visits and 1,400 PNC visits per year respectively, they are privately run and are located in informal settlements. Nazareth is a faith-based tertiary hospital with approximately 8,000 deliveries per year and Jacaranda is a specialized private maternity hospital with approximately 5,700 deliveries per year. All facilities are situated in an urban setting and serve low-resource patients in Nairobi. Each pilot facility signed a memorandum of understanding and a site visit was done to conduct process mapping of patient flow and identify optimal timing for PROM and administrative data collection. At Gertrude’s outreach clinics, patients receive ANC throughout pregnancy and are referred to Jacaranda or Nazareth hospital for the last ANC appointment and delivery. The clinicians and administrative staff of the pilot facilities were trained on data collection, each of the outcome measurements, and VBHC. Additional training on mental health was provided as it was identified as a knowledge gap by staff. Throughout the pilot project, ICHOM supported the facilities through site visits, tele-conferences, and a structured framework designed to support each facility in engaging patients and in data collection and evaluation.

### Participants

The goal was to recruit 200 pregnant women during the third trimester anticipating, based on the experience of local clinicians, that approximately half of the women would be lost to follow up Since the goal of this project was to demonstrate feasibility of a protocol to collect patient-reported outcomes via novel methodology in a low-resource setting, no sample size was calculated.

Inclusion criteria for participants were 1) presenting at the participating clinics for an ANC visit in their third trimester of pregnancy, 2) having access to a mobile phone and personal M-TIBA account, and 3) having comprehension of written English. Exclusion criteria were not owning a mobile phone, gestational age less than 28 weeks or greater than 35 weeks, limited English literacy or having already received prenatal care. Patient enrollment commenced in July 2017 and continued until January 2018. Patients enrolled did not receive any compensation but were given cellphone credit to complete the surveys. Pregnant women were enrolled by PLO at the Gertrudes clinic and written informed consent was obtained. Through the study, the PLO followed up on incomplete responses, missing surveys, or missed visits with a text message. PLOs received standardized training and followed standard protocols (S1 File).

### Data collection

**Fig 2:**
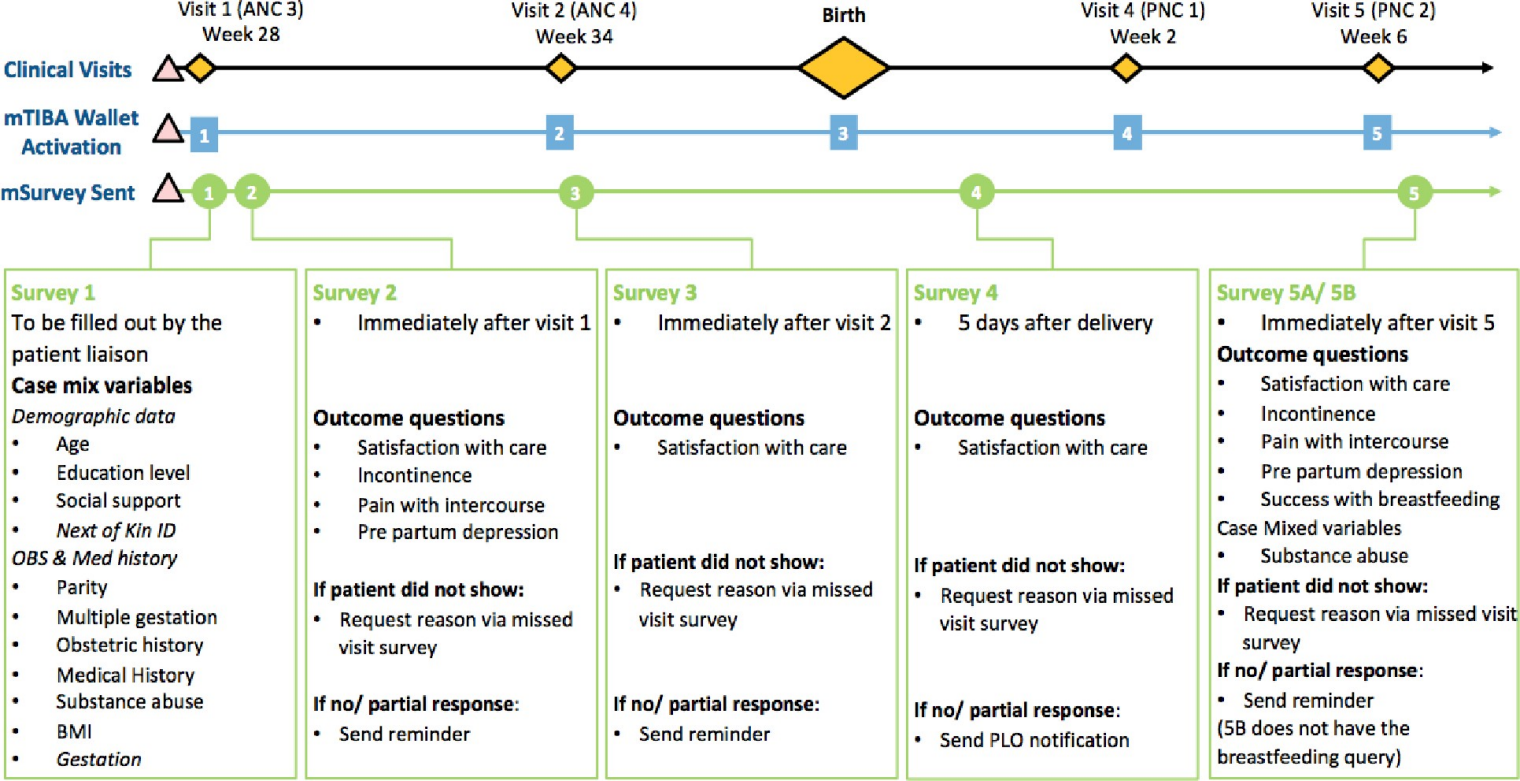
Patient appointment and data flow diagram for women enrolled in the pilot.

Case mix and PROM questions were included in the surveys that were triggered through M-TIBA and sent through the mSurvey platform (Fig 2). Manual data collected by PLOs on women who discontinued their clinical care at participating facilities were captured. Data were collected and stored by PharmAccess. Data from the different databases were linked through unique identifiers ensuring anonymity of the data. Feedback surveys were sent to patients who missed clinic visits or who did not deliver at facilities. Aggregate data were shared with clinicians at the pilot facilities.

### Outcomes

**Fig 3:**
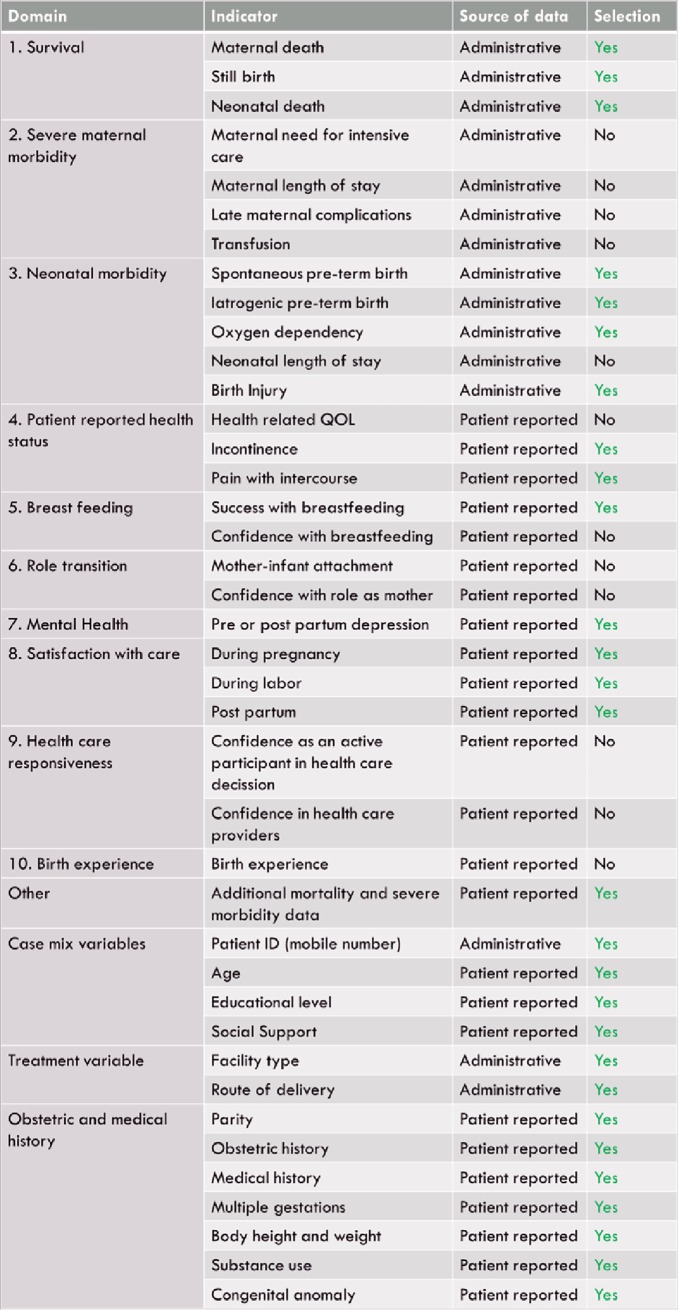
Selection of Standard Set domains and indicators. ICHOM PCB Standard Set domains, indicators and case-mix domains selected for pilot in Nairobi, Kenya. “Yes” indicates selection of the ICHOM indicator for pilot in Kenya.

Of the administratively collected indicators, domains included survival, neonatal morbidity and treatment variables (Fig 3, S2 File). Patient reported indicators spanned the domains of patient reported health status, mental health, breast feeding, satisfaction with care, obstetrical and medical history and case mix variables (Fig 3, S3, S4 and S5 Files).

### Analysis

Following the validation of a subset of data from 15 women, descriptive statistics were generated using SAS software, Version 9.4 (Copyright 2013, SAS Institute Inc., Cary, NC, USA). Sociodemographic characteristics were compared across women using ANOVA and Chi-Square tests, as appropriate.

### Ethics approval

This pilot was approved by the Gertrude's Children's Hospital Ethics Review Board. Data management and analysis was approved by the Harvard Medical School Institutional Review Board.

## Results

### Adaptation of Standard Set to local setting

The ICHOM Standard Set on Pregnancy and Childbirth was adapted for use in Nairobi informal settlements. Of the 26 indicators proposed by ICHOM, 14 were chosen for the pilot. Indicators were excluded if the health care setting did not support delivery of the service or measurement of it (i.e., intensive care, tracking maternal/neonatal length of stay, follow up for late maternal complications, blood transfusion) or if indicators describe concepts not yet widely introduced in the health system (i.e., health-related quality of life, birth experience, confidence in provider, and confidence with breastfeeding, role as a mother, and role as an active participant). All of the case-mix factors were chosen for collection (Fig 3).

The timeline for data collection was adapted to the local context by administering the first survey in the third trimester of pregnancy. It was decided to not distribute a survey during the first PNC visit and only immediately after delivery and after second PNC visit.

### Descriptive data

**Fig 4:**
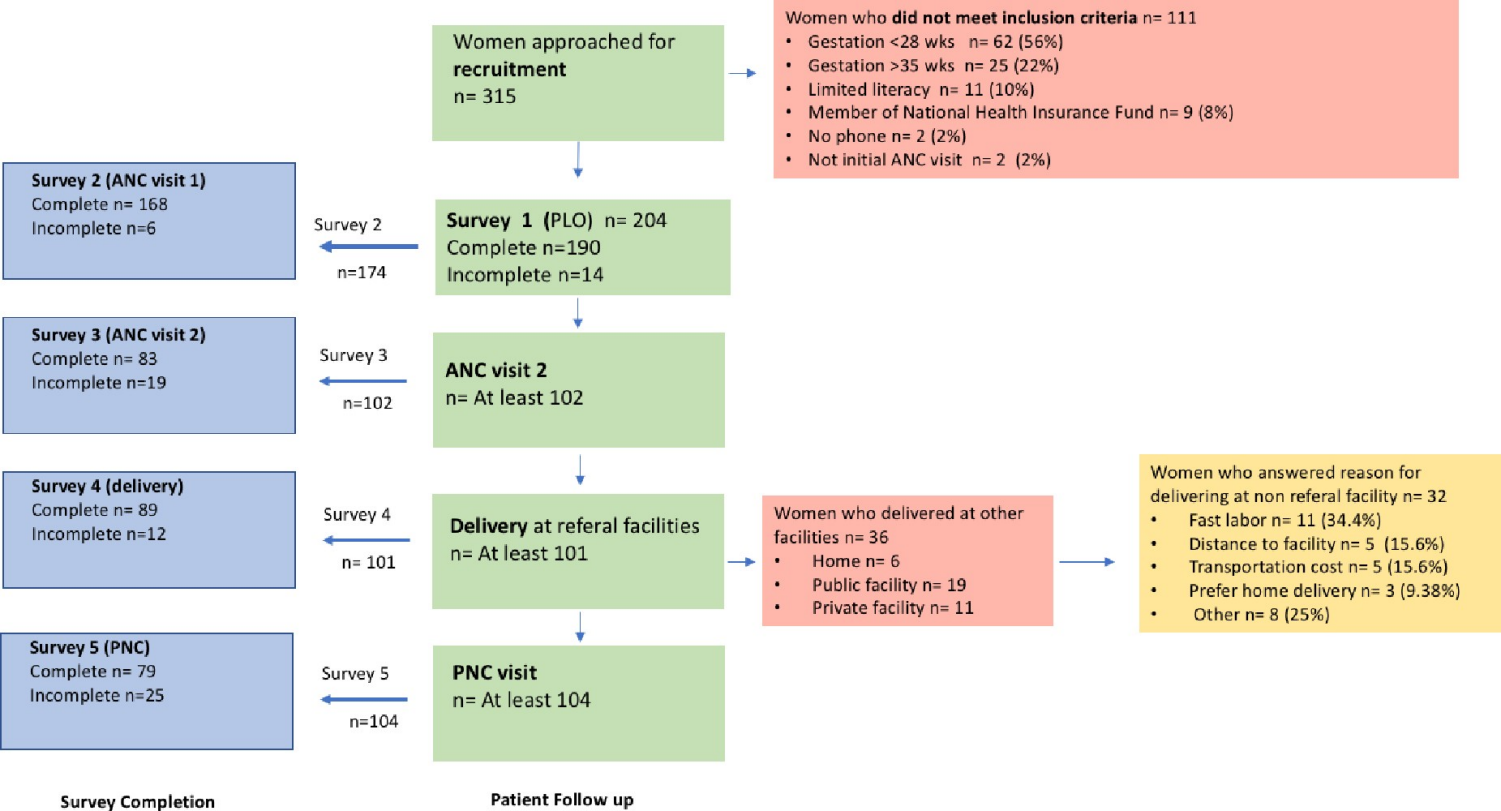
Flow of patients throughout pregnancy and childbirth.

We collected hospital- and patient-reported data on 204 women throughout ANC, delivery, and PNC. A number of women were lost to follow-up (Fig 4) or did not provide a response to each question. Hospital-reported data on delivery type were successfully collected for about half of women (n = 104); 49% of whom had a spontaneous vaginal delivery without complications; 20% had complications, and 31.7% delivered via caesarean. On average, women were 29 years old at the time of recruitment, the average age of the subset that was followed throughout delivery was 26 years old (Table 1). Most women had completed secondary education, reported having two to five people in the community that they could contact if they needed social support, and having no pre-existing health conditions. Overall, gestational age and obstetric history differed by delivery site (*p* = 0.019 and 0.003, respectively). Of the women who reported obstetric history, a higher proportion of women who delivered at a location other than M-TIBA reported a history of past premature delivery or hemorrhage requiring transfusion than women who delivered at an M-TIBA site as planned. However, more women who delivered at M-TIBA reported history of caesarean delivery.

**Table 1:** Patient-reported baseline data.

		All women		Delivered elsewhere		Delivered at M-TIBA	*p*-value
	n	Mean (SD)	n	Mean (SD)	n	Mean (SD)	
Gestational age at beginning of study (weeks)	188	29.1 (1.7)	20	28.4 (0.9)	98	29.3 (1.8)	0.019
Age at time of delivery (years)	181	26.3 (5.0)	20	25.9 (4.5)	91	26.8 (5.2)	0.487
Calculated body mass index (pre-pregnancy)	170	24.6 (4.5)	19	25.2 (5.3)	91	24.0 (3.7)	0.231
		**n (%)**		**n (%)**	** **	**n (%)**	
Education	194		20		99		0.257
None		1 (0.5)		0 (0)		0 (0)	
Primary		36 (18.6)		2 (10.0)		22 (22.2)	
Secondary		113 (58.3)		15 (75.0)		55 (55.6)	
Tertiary (university or equivalent)		44 (22.7)		3 (15.0)		22 (22.2)	
Number of social support contacts in community	204		21		101		0.433
0 persons		26 (12.8)		1 (4.8)		9 (8.9)	
1 person		54 (26.5)		3 (14.3)		28 (27.7)	
2 to 5 persons		119 (58.3)		16 (76.2)		62 (61.4)	
6 to 10 persons		5 (2.5)		1 (4.8)		2 (2.0)	
Reported using any tobacco during pregnancy	191	3 (1.6)	20	0 (0)	99	1 (1.0)	0.652
Reported using drugs during pregnancy	190	11 (5.8)	20	0 (0)	99	6 (6.1)	0.588
Pre-existing health conditions before pregnancy	178		20		90		0.490
None		168 (94.4)		19 (95.0)		88 (97.8)	
Diabetes		3 (1.7)		0 (0)		0 (0)	
Hypertension		7 (3.9)		1 (5.0)		2 (2.2)	
Mental health diagnosis		0 (0)		0 (0)		0 (0)	
Primiparous	193	104 (53.9)	20	7 (35.0)	99	44 (44.4)	0.470
Obstetric history	188		20		91		0.003
Has been pregnant previously		140 (74.5)		11 (55.0)		70 (76.9)	
Premature delivery (<37 weeks)		27 (14.4)		8 (40.0)		9 (9.9)	
Hemorrhage requiring blood transfusion		3 (1.6)		1 (5.0)		1 (1.1)	
Caesarean delivery		15 (8.0)		0 (0)		11 (12.1)	
Loss of pregnancy after 20 weeks		3 (1.6)		0 (0)		0 (0)	
Current pregnancy	192		20		99		1.000
Singleton		187 (97.4)		20 (100.0)		97 (98.0)	
Twins		4 (2.1)		0 (0)		2 (2.0)	
Triplets or more		1 (0.5)		0 (0)		0 (0)	

Total number of respondents for delivery location is equal to the number of women who delivered elsewhere plus those who delivered at an M-TIBA site; note that this reflects missingness. *P*-values were generated via ANOVA or Chi-Square Test, as appropriate.

### Feasibility of patient follow up and data collection

In total, 315 women were approached for recruitment and 204 met the inclusion criteria. The women that did not meet enrollment criteria were excluded as 56% were in the first or second trimester, 22% were over 36 weeks, 10% had limited literacy, 8% had an insurance scheme not compatible with the research methodology, 22% did not own a phone and 2% were not presenting for the initial antenatal care visit. Of all the enrolled women, at least 50% (n = 102) of women returned for a second ANC visit, 49.51% (n = 101) delivered at participating facilities and 50.98% (n = 104) had a PNC visit as reflected by the number of mSurveys triggered by patient clinic registration. Of the women who missed ANC or PNC appointments, some explained that they received care at non-participating facilities while others reported that they were intending to present to clinic at a later time. Of the women that did not deliver at participating referral facility, we were able to track 36 women (Table 2, Fig 4).

**Table 2:** Deliveries at non-participating facilities.

**Hospital-reported data**	**n**	**%**
Alternate delivery location (n = 36)		
Home	6	16.67
Public facility	19	52.78
Private facility	11	30.56
**Patient-reported data**		** **
Reasons for delivering at non-participating facility (n = 32)		
Labor came quickly	11	34.38
Distance to facility	5	15.63
Transport costs	5	15.63
Prefer home delivery	3	9.38
Other	8	25

### Administrative data

Administrative data on maternal and infant mortality and morbidity was successfully collected. There were no maternal deaths during the study and 3 neonatal deaths during the study. The total cost incurred for ANC, delivery, and PNC services was $103, 968 USD for 211 women; the cost per woman varied depending on the number of visits for which they were followed.

### Survey completion

**Fig 5:**
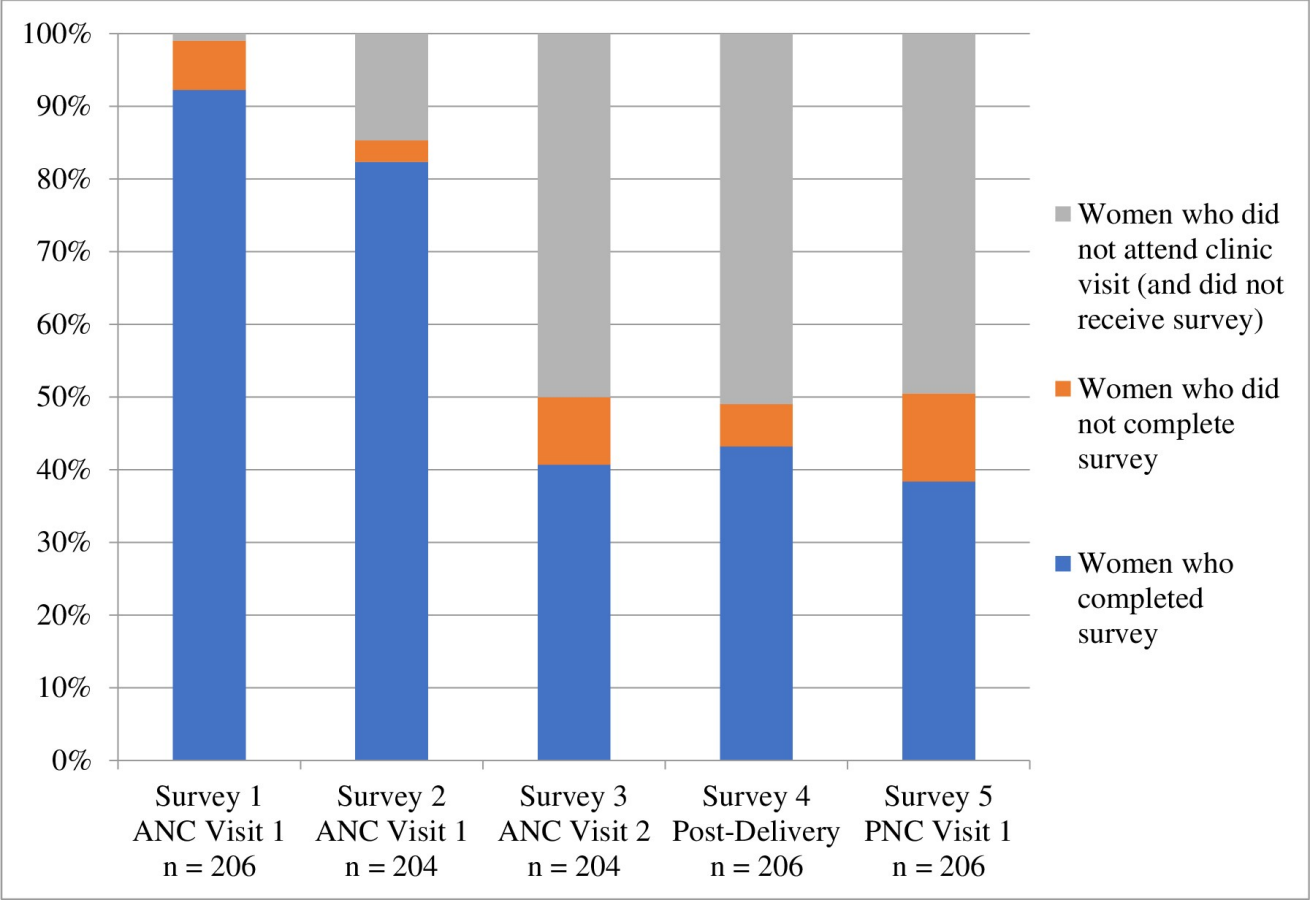
Survey completion. Proportion of completion by survey. Note that women who did not attend clinic may have 1) missed her appointment, 2) sought care at a non-participating clinic, or 3) visited a participating clinic, but did not receive the survey because of a technological error. Technological errors occurred when a payment from the M-TIBA wallet did not trigger a survey, but most of these instances were captured, and team members manually sent the survey, successfully collecting data from those women.

Women’s’ completion of PROM surveys was contingent on attending a participating clinic, at which point payment triggered the survey to be sent to each woman. Completion was highest for the first survey (92%), completed by administrative staff at the hospital. For patient-completed surveys, completion was highest after the first ANC visit (85%), but dropped for the remaining surveys covering a second ANC visit, delivery care, and PNC (Fig 5).

### Patient-reported outcome measures (PROM)

**Fig 6:**
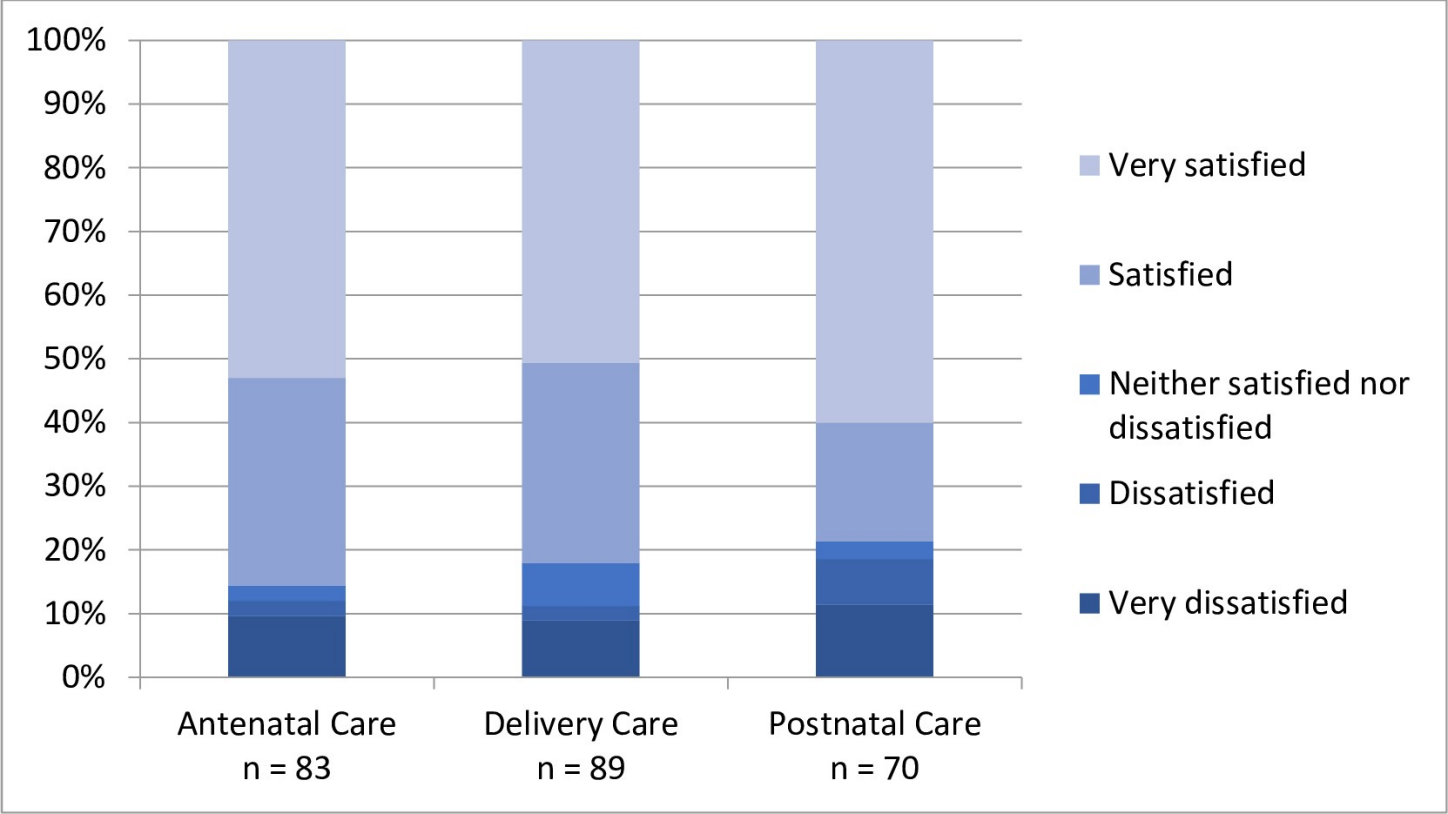
Patient-reported satisfaction with care. Patient-reported satisfaction with care received during antenatal, delivery, and postnatal care periods in response to the following question in Survey 2, 3, and 5: How satisfied are you with the results of your care [during each time period]?.

**Fig 7:**
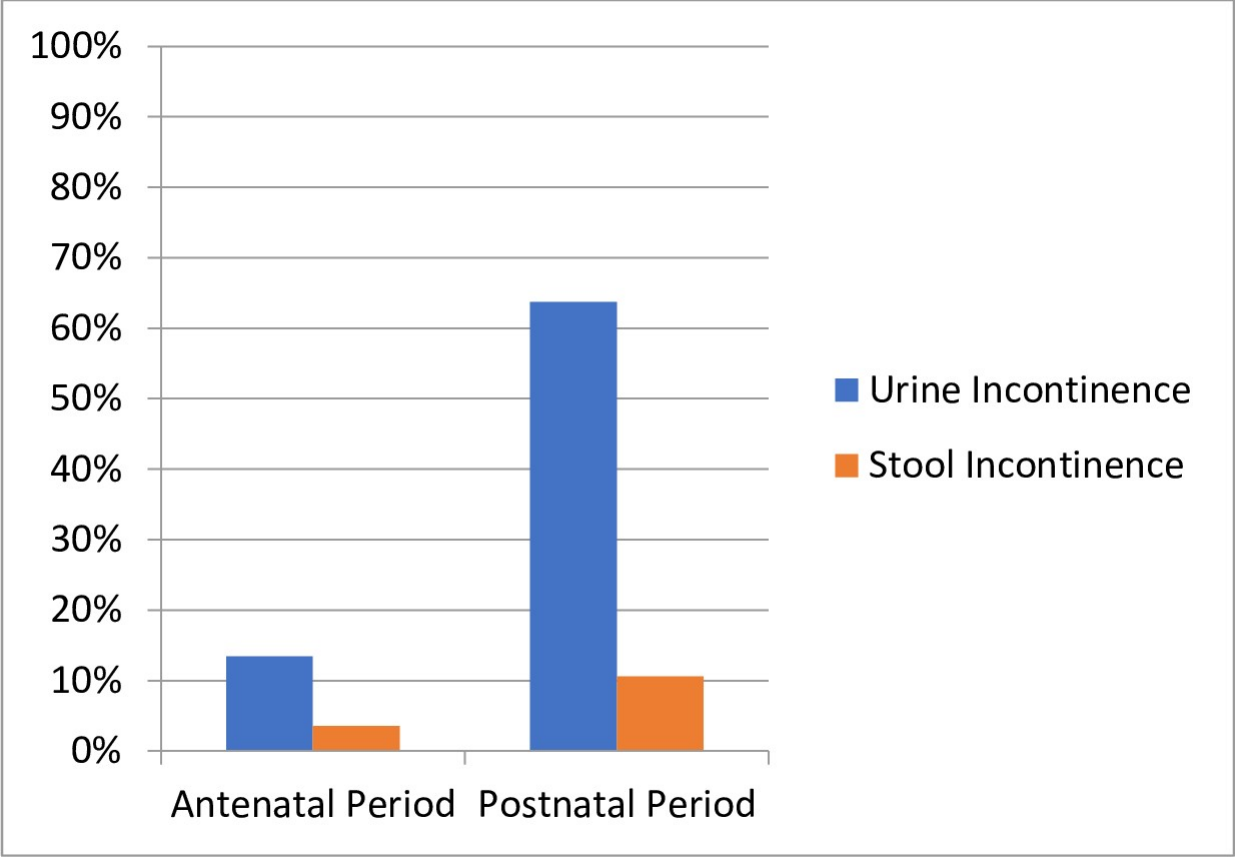
Patient-reported urine and stool incontinence. Patient-reported urine and stool incontinence during antenatal (urine n = 171; stool n = 169) and postnatal (urine n = 193; stool n = 85) care periods in response to Survey 2 and 5 question: In the past month, have you leaked urine, leaked stool, or passed gas by accident? Survey data also captured frequency, quantity, and timing of incontinence, as well as impact on daily life; a very small subset of women reported on these factors.

**Fig 8:**
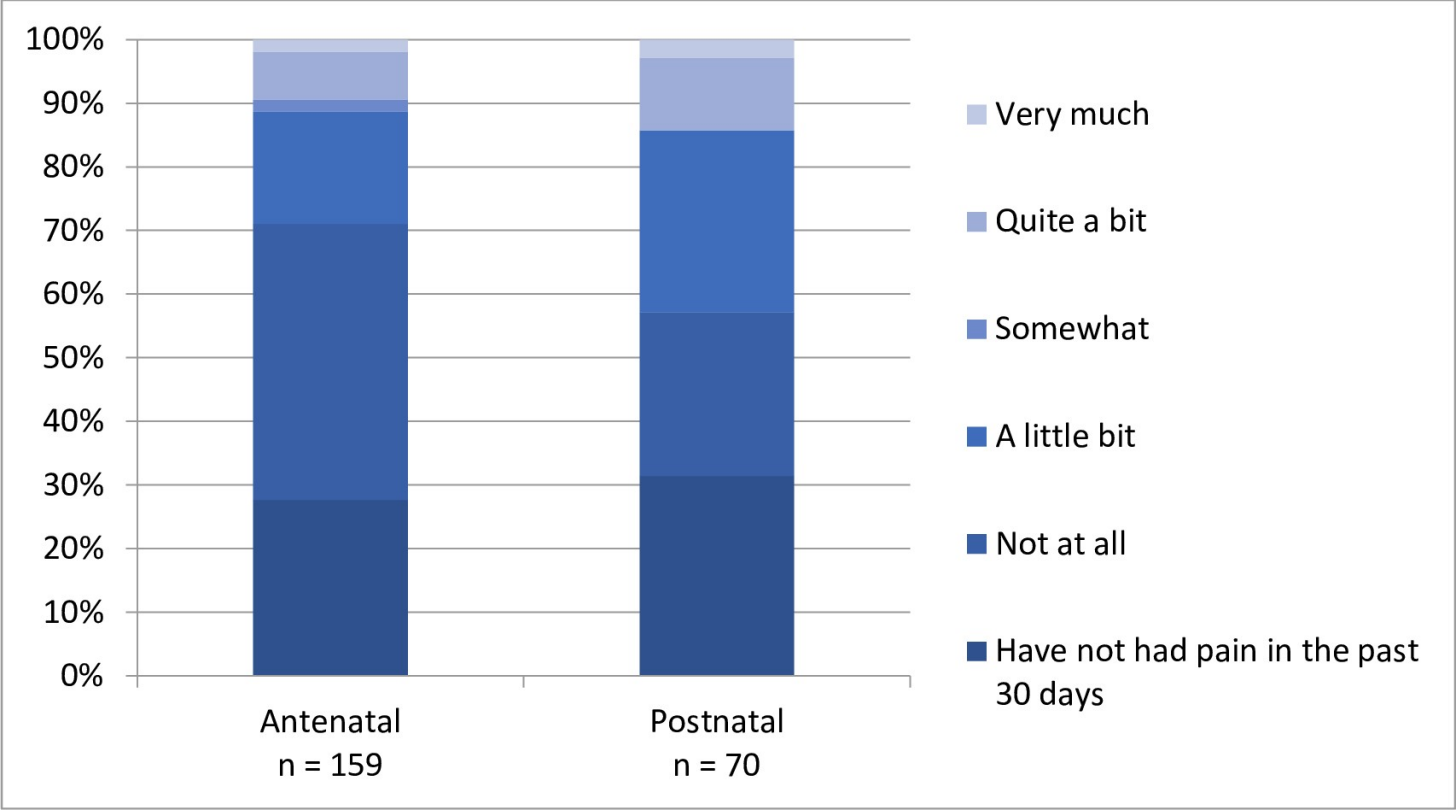
Patient-reported dyspareunia. Patient-reported dyspareunia during antenatal and postnatal care periods in response to Survey 2 and 5 question: In the past 30 days, how much has pain affected your satisfaction with your sex life?.

**Fig 9:**
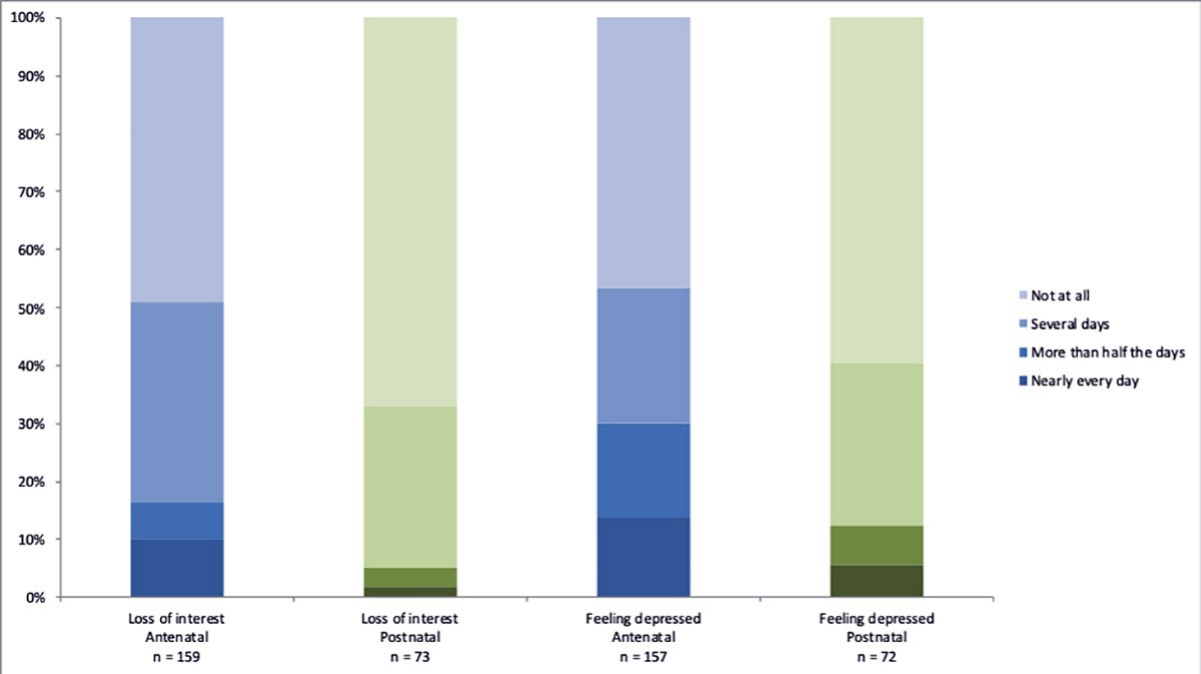
Patient-reported depression symptoms. Patient-reported depression during antenatal and postnatal care periods in response to Survey 2 and 5 question: Over the past two weeks, how often have you been feeling down, depressed, or hopeless? Over the past two weeks, how often have you had little interest or pleasure in doing things?.

Of the women who reported their satisfaction with care, half or more were “very satisfied” with ANC (65.6%), delivery care (50.6%), and PNC (60%) (Fig 6). With regard to clinical outcomes, more women reported both urinary and stool incontinence in PNC compared to ANC (Fig 7). During both ANC and PNC periods, most women reported that they did not experience dyspareunia (Fig 8). Nearly half of women reported symptoms of depression during ANC and PNC (Fig 9). During the PNC visit, of 70 respondents, most women were exclusively breastfeeding (85.7%), some were choosing a combination of breastfeeding and formula or other liquids (11.4%), and few were using formula or other liquids exclusively (2.9%).

## Discussion

This pilot demonstrates the feasibility of collecting longitudinal data, including PROMs, through ANC, delivery, and PNC from women in a low-resource setting utilizing mobile technology. These data enable researchers and policymakers to understand vulnerable patients’ needs, care-seeking behavior, and self-reported outcomes. This is critical to informing effective policy to drive progress toward SDG 3.

### Adaptation of Standard Set to a low-resource setting

Adaptation of the ICHOM PCB Standard Set to the local cultural setting was essential prior to pilot implementation; questions on ethnicity were removed and questions on social support were modified. Mental health, was identified as a gap in knowledge by hospital staff and extra training was provided. The lack of awareness among providers around mental health is consistent with our results in which none of the women reported having a history of mental illness, and yet nearly half of them reported symptoms of depression during ANC and PNC.

### Utilization of care

The World Health Organization reports that 92% of women in Kenya attended at least one ANC visit in 2015 [35], and the 2014 Demographic and Health Survey (DHS) indicates that only 58% of women attend the recommended four ANC visits, 61.2% deliver at a health care facility by a skilled provider, and 52.9% receive a PNC visit within the first 2 days after delivery [36]. In comparison, among those enrolled in our pilot, at least 50% attended two ANC visits at a participating facility, 56% delivered at referral hospitals and at least 38% attended PNC six weeks post-delivery. Our study showed that women’s attendance to clinics decreases over time; a small sub-group reported receiving care at a non-participating facility, and some women report distance, transportation cost or precipitous delivery as the cause. A 2009 study in rural Tanzania showed that bypassing care facilities for childbirth is frequent and often motivated by patients’ concerns about quality of care [7]. Evidence suggests that utilization of high quality care can reduce mortality[37] and PROMs enable measuring and improving quality. Mobile data collection may also be useful to understand barriers to seeking or reaching care. Additional data are needed to better understand access, utilization, and quality of PCB services.

### Patient-reported exposures

A cross-sectional study on morbidity of 3,145 pregnant women in Kenya found that 2% of women report use of alcohol, tobacco, sedatives and inhalants, and 21.7% report intimate partner violence[6]. In comparison, 1.57% of women in our pilot reported using tobacco and 5.79% reported drug use during pregnancy. Furthermore, McCauley et al. reported that 4.5% of women in Kenya experience thoughts of self-harm and 12.3% had symptoms of postnatal depression [6]. In our study, 33% of women report little interest or pleasure in doing things, and 40% feeling down, depressed, or hopeless in the post-natal period. Higher prevalence in our study may relate to differences in definitions of outcomes or true increases revealed through the anonymity of the mSurvey.

### Patient-reported outcomes

In our study, 13.5% of women reported symptoms of urinary incontinence during the ANC period. In contrast, a recent study in Kenya found that 1.6% had urinary incontinence[6]. The difference could be due to women being more willing to report this anonymously or due to our population having larger incidence of incontinence. Answering sensitive PROM questions via text messages may facilitate honest answers more easily than traditional in-person interviews or household surveys. In our study, women reported high levels of satisfaction with the care provided throughout pregnancy. Interestingly, a study in Tanzania showed that the characteristics of health facilities did not influence women’s ratings of quality but instead her expectations and experiences influenced her perception[38]. In the future, this tool could evolve from use in data collection to use in care delivery by incorporating algorithms to trigger clinical follow up upon particular patient responses.

### Quality improvement and policy implications

Data were reviewed every three months by clinicians at each participating facility to discuss gaps in referrals, losses to follow up, and patient outcomes with the overarching goal of identifying areas for improvement. Facilities are mandated to report outcomes to the government, and using data for benchmarking and improvement instead of with a punitive intent could encourage transparency and increase reporting accuracy, ultimately driving effective policy.

Quality of PCB care is especially critical given that women of reproductive age contribute to national economic growth. In Kenya, the value of lost output from mortality amenable to high-quality health care resulted in a projected cumulative loss of $57,633 million USD during 2015–30 and a potential economic loss of 2.1% of the country’s GDP by 2030[39]. Since PCB is one of the most common reasons women seek medical care, improving the quality of PCB care is critical to sustaining the economy.

### Feasibility and generalizability

Using a mobile platform, we were able to capture data on PROM of women with survey completion rates that were highest for the ANC period at 85% and dropped to 38% in the PNC period. Socio-demographic factors have been associated with less access to care and increased maternal complications [40]. Mobile technology facilitated outreach to women in informal settlements while providing them anonymity for reporting of health outcomes. The participating facilities were already part of a quality improvement program; while targeting hospitals championing quality improvement facilitate pilot implementation, sites in which a culture of quality has not been established are likely to require additional support. Despite mobility and active decision making, many women were tracked with the mobile platform and PLOs who followed up those who missed appointments. In scaling up this methodology, training PLOs would be highly beneficial as their role created awareness among women about their health seeking behaviors and empowered them to report on their health outcomes. The PLOs importance is consistent with literature that shows that accompaniment of pregnant women is associated with increases in institutional delivery, satisfaction with care and access to postnatal care[41, 42].

As the pilot progressed, we identified questions requiring further refining and encountered technological difficulties that resulted in missing surveys and exclusion of women enrolled in the national insurance funds due to software incompatibility. While we don’t anticipate this introduced bias to our results, it highlights the importance of continuously troubleshooting robust mobile platforms to optimize data collection. Our platform successfully linked administrative data from the clinics with the mSurvey, completeness and accuracy of data collection will further help evaluate the feasibility of this methodology.

### Future direction

The next steps are to scale up this mobile platform for PROM collection, reduce the costs of implementation and ensure its sustainability. The scale-up of this methodology should include a wider population of women, including non-English speakers and those attending public facilities and in rural areas. As diversity and sample size increases, sub-analyses may improve understanding of how socio-economic factors differ between women who do not deliver at their referral facility. Increased ownership from local practitioners and use of automated real-time data dashboards would facilitate scale up and effective identification of areas for quality improvement initiatives. Identifying policy-makers interested in value-based health care to incentivize transparent comparison of PROM for benchmarking will be key to facilitate adequate allocation of resources. This model presents an excellent opportunity to develop a business model that combines quality improvement and value-based financing and attract national and international funds in maternal and neonatal health. Furthermore, this platform could be expanded to follow up infants and to increase the effectiveness of community health workers following up families and increasing their access to high quality care.

### Limitations

Due to financial constraints, participation was limited to women who owned a cellphone and comprehend written English. However, English is one of two official languages and mobile phone penetration is estimated at 95.1%[43]. Enrollment was limited to women in the third trimester and as a result 62.56% of women excluded from the study were ineligible because of an earlier gestational age (Fig 4). Future implementation must have broader inclusion criteria. Despite PLO’s attempts to follow up patients that did not return to clinic this was not possible in all cases.

## Conclusion

Every woman has the right to the highest standard of health and well-being, these are central to the 2030 Agenda for Sustainable Development. The need to standardize the measurement of maternal morbidity has been widely recognized [44–46]. We have demonstrated the role that technology can play in measurement of standardized outcomes in low resource settings and a rising opportunity for new business models around outcomes based financing.

## Supporting information

S1 File Patient liaison officer training manual. (DOCX)

S2 File Survey #1. Survey completed by patient liaison officer on first visit containing demographic and baseline clinical history information.
(DOCX)

S3 File Survey #2 and #3. Survey on patient reported outcomes completed by patient after first and second ante-natal care visit.
(DOCX)

S4 File Survey #4. Survey on patient reported outcomes completed by patient after delivery.
(DOCX)

S5 File Survey #5. Survey on patient reported outcomes completed by patient after post-natal care visit.
(DOCX)
