# Supplementary material for: Implementation of an international standardized set of outcome indicators in pregnancy and childbirth in Kenya: Utilizing mobile technology to collect patient-reported outcomes
Source: PLoS One. 2019 Oct 16;14(10):e0222978. doi: 10.1371/journal.pone.0222978 (PMC6795527; doi:10.1371/journal.pone.0222978)
Supplement: S1 File — (DOCX) [file pone.0222978.s001.docx]

**Supplemental material**

**S1. Patient Liaison Officer Training Manual**

The following document is a guide to support you in your role as Patient Liaison Officer to those patients enrolled in this pilot scheme.

PharmAccess have partnered with the International Consortium for Health Outcomes Measurement (ICHOM) to pilot data collection for women during pregnancy and childbirth. Using MTIBA, we intend to collect clinical and demographic data, as well as a mobile survey to collect the patient reported outcomes. These surveys and the patient responses will be collected via SMS. Data will be collected from each woman enrolled in the pilot scheme at specified time-points during their pregnancy and following childbirth.

On the following pages are guidelines to support you with your patient encounters, detailing how to enrol patients to the pilot scheme, common questions you may face and how to best respond to them, scenarios where you may need to refer patients for additional care and support. Chapter 6 has a template of the forms you should complete at each encounter with the patient, as well as useful contact numbers to support you in your role.

**Chapter 1: Enrolling patients**


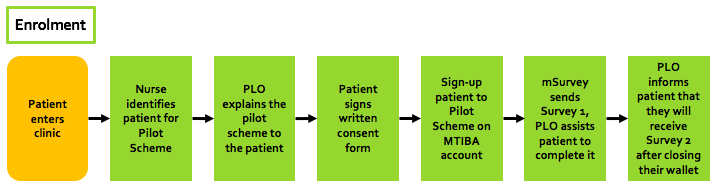


The nurse at your facility will identify eligible women to enrol to the pilot scheme:

- Women who are in their third trimester of pregnancy
- Have comprehension of English
- Have a mobile phone and are registered on the Smiles Program and MTIBA

**What information should be shared with the patient?**

Below is an example of the information you can relay to the patient at enrolment

This is a pilot scheme which aims to identify your outcomes during your pregnancy, during birth and in the weeks following. The objective is to improve healthcare of individual women in the pilot and the community. You will be sent a series of questions that will ask you about:

1. Your satisfaction with the care you received during your visit to the clinic
2. Incontinence( Involuntary leaking of urine or stool)
3. Your mental wellbeing pre- and post-partum (to screen for depression)
4. Whether you experience pain during sexual intercourse
5. Success with breastfeeding

We will gather this information from you after each visit and will send a reminder to complete the survey after two days. The information you supply will enable us to guide you, particularly where you may require referral or additional support from your clinicians. We will also use this information to identify where improvements to the ANC, delivery and PNC healthcare services can be made. All information that you share via MTIBA, the SMS surveys and when discussing with your PLO will be confidential.

If you have any difficulty completing the questionnaire, or require support, you are advised and welcome to contact your PLO, who will guide you through the next steps.

After establishing the patients’ interest in joining the pilot, please share the consent form with them and request they sign, before activating the ICHOM scheme on their MTIBA wallet.

**Chapter 2: The questionnaires**

Once enrolled and the MTIBA wallet has been activated, each expectant mother will be sent a series of questions following each visit to a facility. The timeline and corresponding questionnaires can be found in the Supplemental Information

Each survey will be sent to immediately after the wallet is closed and billing is complete, with the exception of **delivery**, whereby the survey will be sent 5 days following the registered delivery date.

**Chapter 3: Patients who do not respond to the survey**


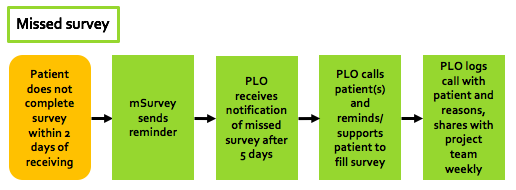


Follow-up will be required for patients who do not respond to their surveys or who only partially complete the survey. You will receive a notification from mSurvey with the numbers of those patients, for you to contact. All correspondence should be logged in Form 1 (see Chapter 6).

**What to do if a patient has not/only partially responded to a survey?**

Call the patient and confirm they are enrolled in the scheme.

Confirm that they have not completed the survey and proceed to ask why.

Common responses may include:

1. Forgot to complete Give a reminder to complete and ask whether
2. Did not have the time they require any support in completing the

questions

1. Did not understand Review the question with the patient, explaining any terms that were unclear
2. Do not wish to complete Log the reason(s) why they no longer wish

/ participate to participate. Send notification to MTIBA to remove from the pilot scheme

Log the correspondence with the patients, what action was taken and send this information to the shared database. Fill the online correspondence Form 1 and share this on a weekly basis Ramona Koech [r.koech@pharmaccess.or.ke]

**Chapter 4: Patients who miss appointments**


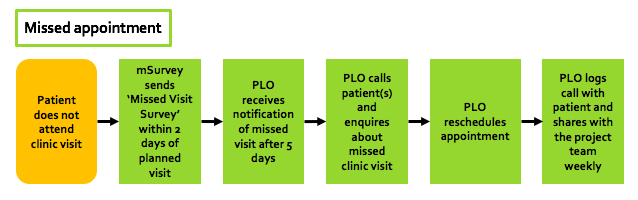


Those who miss their scheduled appointment will be sent a ‘Missed Visit Survey’ within two days (of the missed date). This survey will ask the patient to select a reason for missing the appointment and prompting them to attend the clinic.

If the patient has not responded within five days of receiving the ‘Missed Visit Survey,’ the project team will share the contact numbers of those patients, for you to contact.

Please note the patient’s reason for missing their appointment on Form 2 (including, but not limited to: illness, did not have transport, appointment was not due).

If the patient does not attend the hospital for delivery, please note the reasoning, such as:

- Delivered at home
- Delivered at a different facility
- Did not have mobile phone with them at time of delivery
- Patient has passed away
- Not yet delivered

**Chapter 5: Follow-up on patients with low outcome scores**

**
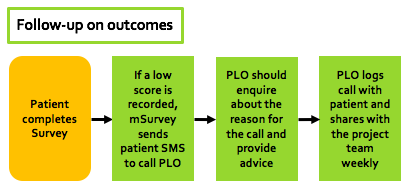
**

Each patient will receive the following questions at two time points, during this pilot: during their third trimester and 42 days’ post-partum.

1. Satisfaction with the care
2. Incontinence
3. Mental wellbeing pre- and post-partum (to screen for depression)
4. Pain during intercourse
5. Success with breastfeeding

If the patient receives a low-score for any of the questions in the survey, they will be prompted to call you, the PLO officer, via an SMS from mSurvey. Detailed below, is a guideline of how to support and where to refer the patient, depending on their score for each survey.

First enquire about why the patient has called and how you can support them. If they have called because they received an SMS prompting them to call you. Review each survey and their responses over the phone (see Table 1). If you are unsure or need clarification please contact a member of the project team.

Table 1. Follow-up support guide.

| **Questionnaire** | **Score** | **How to respond** |
| --- | --- | --- |
| Satisfaction with care | **Less than or equal to 2** | 1. Ask the patient which facility they attended. 2. Log the reasoning for the low satisfaction with care. Let the patient know that this information will be fed back to the facility. 3. Share this with member of the project team who will feedback to the facility. |
| Incontinence | Response = **Yes** to the incontinence screening question | 1. Following the screening question, and if they responded yes, the patient will have received a series of question (see Chapter 2). 2. Ask the patient whether they have had this reviewed by a clinician at their last appointment? 3. If yes, determine whether they have received any counselling or medication to treat this. 4. If no, refer to the nurse at the clinic who will contact with the project team. |
| Mental wellbeing | T**otal score of 3 or more** for both questions | 1. Ask the patient to provide additional detail regarding their mood over the last 7 days. 2. Determine whether they have reviewed this with the clinician, during their last appointment. Identify whether any counselling/ referral for further treatment was received. Log this on Form 3. 3. If they answer ‘Yes’ to having any mental health issues, refer to their nurse at the clinic, who should refer them to the social worker for counselling. |
| Pain during sexual intercourse | Score **equal to or more than 2** | 1. Ask the patient whether they have reviewed this with the clinician, during their last appointment. 2. Identify whether any counselling/ referral for further treatment was received. Log their response. 3. If they answer ‘Yes’ to experiencing pain, refer to their nurse at their clinic. |
| Success with breastfeeding |  | 1. Ask the patient to detail any challenges they are having in breastfeeding. 2. Identify whether they have received any counselling or support in their previous appointment. 3. If they are facing difficulty in breastfeeding, refer to their nurse at the clinic, who should provide additional counselling. |

**Chapter 6: Forms and contact numbers**

The following forms are templates of those that you will fill on your tablet. They are designed for you to track your correspondence with each patient. Please keep this confidential information in a secure place, only sharing the forms with Ramona Koech via email [r.koech@pharmaccess.or.ke]

Form 1: Incomplete survey

Form 2: Missed appointment

Form 3: Following up on outcomes

**Useful contact numbers;**

Should you require any support, please contact the following:

1. Technical support:
   1. CarePay
   2. mSurvey
2. PharmAccess:

**Form 1: Incomplete survey**

| **PLO ID** | **Date** | **Patient number** | **Age and gestation** | **Please specify what question(s) were not completed** | **Please specify whether the survey was incomplete/ not completed** | **Please note the reasons given for not completing the survey:**   1. **Forgot to complete** 2. **Did not have time** 3. **Did not understand question** 4. **Do not wish to complete** 5. **Other, please specify** | **Please note what action has been taken (i.e. patient will complete, opt-out etc.)** |
| --- | --- | --- | --- | --- | --- | --- | --- |
|  |  |  |  |  |  |  |  |
|  |  |  |  |  |  |  |  |
|  |  |  |  |  |  |  |  |
|  |  |  |  |  |  |  |  |
|  |  |  |  |  |  |  |  |
|  |  |  |  |  |  |  |  |
|  |  |  |  |  |  |  |  |
|  |  |  |  |  |  |  |  |
|  |  |  |  |  |  |  |  |
|  |  |  |  |  |  |  |  |
|  |  |  |  |  |  |  |  |
|  |  |  |  |  |  |  |  |
|  |  |  |  |  |  |  |  |
|  |  |  |  |  |  |  |  |
|  |  |  |  |  |  |  |  |
|  |  |  |  |  |  |  |  |
|  |  |  |  |  |  |  |  |

**Form 2: Missed appointment**

| **PLO ID** | **Date** | **Age and gestation** | **Patient number** | **Please specify which appointment was missed** | **Please note the reasons given for not attending their last appointment** | **Please note what action has been taken (i.e. patient will complete, opt-out etc.)** |
| --- | --- | --- | --- | --- | --- | --- |
|  |  |  |  |  |  |  |
|  |  |  |  |  |  |  |
|  |  |  |  |  |  |  |
|  |  |  |  |  |  |  |
|  |  |  |  |  |  |  |
|  |  |  |  |  |  |  |
|  |  |  |  |  |  |  |
|  |  |  |  |  |  |  |
|  |  |  |  |  |  |  |
|  |  |  |  |  |  |  |
|  |  |  |  |  |  |  |
|  |  |  |  |  |  |  |
|  |  |  |  |  |  |  |
|  |  |  |  |  |  |  |
|  |  |  |  |  |  |  |
|  |  |  |  |  |  |  |
|  |  |  |  |  |  |  |
|  |  |  |  |  |  |  |
|  |  |  |  |  |  |  |
|  |  |  |  |  |  |  |
|  |  |  |  |  |  |  |
|  |  |  |  |  |  |  |

**Form 3: Following up on outcomes**

| **PLO ID** | **Date** | **Patient number** | **Please specify what outcomes were discussed**   1. **Satisfaction with care** 2. **Incontinence** 3. **Mental health** 4. **Pain during sexual intercourse** 5. **Success with breastfeeding** | **Please note what was discussed and what action has been taken** |
| --- | --- | --- | --- | --- |
|  |  |  |  |  |
|  |  |  |  |  |
|  |  |  |  |  |
|  |  |  |  |  |
|  |  |  |  |  |
|  |  |  |  |  |
|  |  |  |  |  |
|  |  |  |  |  |
|  |  |  |  |  |
|  |  |  |  |  |
|  |  |  |  |  |
|  |  |  |  |  |
|  |  |  |  |  |
|  |  |  |  |  |
|  |  |  |  |  |
|  |  |  |  |  |
|  |  |  |  |  |
|  |  |  |  |  |
|  |  |  |  |  |
|  |  |  |  |  |
