# Supplementary material for: Implementation of an international standardized set of outcome indicators in pregnancy and childbirth in Kenya: Utilizing mobile technology to collect patient-reported outcomes
Source: PLoS One. 2019 Oct 16;14(10):e0222978. doi: 10.1371/journal.pone.0222978 (PMC6795527; doi:10.1371/journal.pone.0222978)
Supplement: S2 File — Survey completed by patient liaison officer on first visit containing demographic and baseline clinical history information. (DOCX) [file pone.0222978.s002.docx]

**S2. Survey #1.** Survey completed by patient liaison officer on first visit containing demographic and baseline clinical history information

| **Variable ID:** | AGE |
| --- | --- |
| **Variable:** | Age |
| **Definition:** | Age at time of delivery |
| **Supporting Definition:** | N/A |
| **Inclusion Criteria:** | All women |
| **Timing:** | 42 days postpartum |
| **Reporting Source:** | Administrative |
| **Type:** | Numerical |
| **Response Options:** | Numerical value |
| **Variable ID:** | EDUCATION |
| **Variable:** | Education level |
| **Definition:** | Please indicate the highest level of schooling completed. |
| **Supporting Definition:** | N/A |
| **Inclusion Criteria:** | All women |
| **Timing:** | Entry to prenatal care |
| **Reporting Source:** | Patient-reported |
| **Type:** | Single answer |
| **Response Options:** | 0 = None  1 = Primary  2 = Secondary  3 = Tertiary (university or equivalent) |
| **Variable ID:** | SIMSS |
| **Variable:** | Social Support |
| **Definition:** | How many people do you have near you that you can readily count on for help in time of difficulty such as to watch over children or pets, give rides to the hospital or store, or help when you are sick? |
| **Supporting Definition:** | The SIMSS |
| **Inclusion Criteria:** | All women |
| **Timing:** | Entry to prenatal care |
| **Reporting Source:** | Patient-reported |
| **Type:** | Single answer |
| **Response Options:** | 0 = 0  1 = 1  2 = 2 - 5  3 = 6 - 10 |
| **Variable ID:** | PARITY |
| **Variable:** | Parity |
| **Definition:** | Have you given birth before? This includes both vaginal births and Cesarean sections (operations to remove your baby from your abdomen). Please do not count miscarriages or births that happened before 20 weeks (5 months) of pregnancy. |
| **Supporting Definition:** | N/A |
| **Inclusion Criteria:** | All women |
| **Timing:** | Entry to prenatal care |
| **Reporting Source:** | Patient-reported |
| **Type:** | Single answer |
| **Response Options:** | 0 = No  1 = Yes |
| **Variable ID:** | OBHX |
| **Variable:** | Obstetric history |
| **Definition:** | If you have been pregnant before, have you experienced any of the following in previous pregnancies? Please mark all that apply. |
| **Supporting Definition:** | N/A |
| **Inclusion Criteria:** | All women |
| **Timing:** | Entry to prenatal care |
| **Reporting Source:** | Patient-reported |
| **Type:** | Multiple answers |
| **Response Options:** | 0 = This is my first pregnancy  1 = A baby born early, more than 3 weeks before his or her due date  2 = Bleeding so much during pregnancy, birth, or after giving birth that you needed to be given blood  3 = A cesarean section (operation to remove your baby through your abdomen)  4 = Loss of a pregnancy after 20 weeks (5 months) of pregnancy |
| **Variable ID:** | MEDHX |
| **Variable:** | Medical history |
| **Definition:** | BEFORE you got pregnant, did a doctor, nurse, or other health worker tell you that you had any of the following health conditions? Please mark all that apply. |
| **Supporting Definition:** | N/A |
| **Inclusion Criteria:** | All women |
| **Timing:** | Entry to prenatal care |
| **Reporting Source:** | Patient-reported |
| **Type:** | Multiple answers |
| **Response Options:** | 0 = None  1 = Diabetes  2 = High blood pressure or hypertension  3 = A mental health disorder such as depression, anxiety, bipolar disorder or schizophrenia |
| **Variable ID:** | MULTGEST |
| **Variable:** | Multiple gestations |
| **Definition:** | Are you pregnant with: |
| **Supporting Definition:** | N/A |
| **Inclusion Criteria:** | All women |
| **Timing:** | Entry to prenatal care |
| **Reporting Source:** | Patient-reported |
| **Type:** | Single answer |
| **Response Options:** | 1 = One baby  2 = Two babies (twins)  3 = Three or more babies (triplets or higher) |
| **Variable ID:** | HEIGHT |
| **Variable:** | Body height |
| **Definition:** | How tall are you? |
| **Supporting Definition:** | Indicate height in centimeters or inches. Also indicate units of height. Height and weight are used to calculate BMI. |
| **Inclusion Criteria:** | All women |
| **Timing:** | Entry to prenatal care |
| **Reporting Source:** | Patient-reported |
| **Type:** | Numeric value |
| **Response Options:** | Numeric value of height in metric or imperial system |
| **Variable ID:** | HEIGHTUNIT |
| **Variable:** | Body height units |
| **Definition:** | Height units |
| **Supporting Definition:** | N/A |
| **Inclusion Criteria:** | All women |
| **Timing:** | Entry to prenatal care |
| **Reporting Source:** | Patient-reported |
| **Type:** | Single answer |
| **Response Options:** | 1 = centimeters  2 = inches |
| **Variable ID:** | WEIGHT |
| **Variable:** | Body weight |
| **Definition:** | How much did you weigh IMMEDIATELY before your pregnancy? |
| **Supporting Definition:** | Indicate weight in kilograms or pounds. Also indicate units of weight. Height and weight are used to calculate BMI. |
| **Inclusion Criteria:** | All women |
| **Timing:** | Entry to prenatal care |
| **Reporting Source:** | Patient-reported |
| **Type:** | Numeric value |
| **Response Options:** | Numeric value of weight in metric or imperial system |
| **Variable ID:** | WEIGHTUNIT |
| **Variable:** | Body weight units |
| **Definition:** | Weight units |
| **Supporting Definition:** | N/A |
| **Inclusion Criteria:** | All women |
| **Timing:** | Entry to prenatal care |
| **Reporting Source:** | Patient-reported |
| **Type:** | Single answer |
| **Response Options:** | 1 = kilograms  2 = pounds |
| **Variable ID:** | SUBUSETOB |
| **Variable:** | Substance use tobacco |
| **Definition:** | Tobacco use during pregnancy |
| **Supporting Definition:** | N/A |
| **Inclusion Criteria:** | All women |
| **Timing:** | 42 days postpartum |
| **Reporting Source:** | Administrative |
| **Type:** | Single answer |
| **Response Options:** | 0 = No  1 = Yes |
| **Variable ID:** | SUBUSEDRUG |
| **Variable:** | Substance use drug |
| **Definition:** | Drug use complicating the puerperium |
| **Supporting Definition:** | N/A |
| **Inclusion Criteria:** | All women |
| **Timing:** | 42 days postpartum |
| **Reporting Source:** | Administrative |
| **Type:** | Single answer |
| **Response Options:** | 0 = No  1 = Yes |
| **Variable ID:** | SUBUSEALC |
| **Variable:** | Substance use alcohol |
| **Definition:** | Alcohol use complicating the puerperium |
| **Supporting Definition:** | N/A |
| **Inclusion Criteria:** | All women |
| **Timing:** | 42 days postpartum |
| **Reporting Source:** | Administrative |
| **Type:** | Single answer |
| **Response Options:** | 0 = No  1 = Yes |
