# Supplementary material for: Implementation of an international standardized set of outcome indicators in pregnancy and childbirth in Kenya: Utilizing mobile technology to collect patient-reported outcomes
Source: PLoS One. 2019 Oct 16;14(10):e0222978. doi: 10.1371/journal.pone.0222978 (PMC6795527; doi:10.1371/journal.pone.0222978)
Supplement: S3 File — Survey on patient reported outcomes completed by patient after first and second ante-natal care visit. (DOCX) [file pone.0222978.s003.docx]

**S3. Survey #2 and #3.** Survey on patient reported outcomes completed by patient after first and second ante-natal care visit

| **Variable:** | Incontinence screening question |
| --- | --- |
| **Definition:** | In the past month, have you leaked urine, leaked stool or passed gas by accident? |
| **Type:** | Single Answer |
| **Response Options:** | 1 = Yes, I leaked urine  2 = Yes, I leaked stool or passed gas by accident  3 = No, I did not leak urine, leak stool, or pass gas by accident |
| **Variable:** | Question 1 of the ICIQ-SF |
| **Definition:** | How often do you leak urine? |
| **Inclusion Criteria:** | *women scoring a 1 on INCONTSCREEN |
| **Type:** | Single Answer |
| **Response Options:** | 0 = never  1 = about once a week or less often  2 = two or three times a week  3 = about once a day  4 = several time a day  5 = all the time |
| **Variable:** | Question 2 of the ICIQ-SF |
| **Definition:** | We would like to know how much urine you think leaks. How much urine do you usually leak (whether you wear protection or not)? |
| **Type:** | Single Answer |
| **Response Options:** | 0 = none  2 = a small amount  4 = a moderate amount  6 = a large amount |
| **Variable:** | Question 3 of the ICIQ-SF |
| **Definition:** | Overall, how much does leaking urine interfere with your everyday life? Please select a number between 0 (not at all) and 10 (a great deal). |
| **Type:** | Single Answer |
| **Response Options:** | 0 = not at all  1 = 1  2 = 2  3 = 3  4 = 4  5 = 5  6 = 6  7 = 7  8 = 8  9 = 9  10 = a great deal |
| **Variable:** | Question 4 of the ICIQ-SF |
| **Definition:** | When does urine leak?(Please tick all that apply to you). |
| **Type:** | Single Answer |
| **Response Options:** | Never - urine does not leak  Leaks before you can get to the toilet  Leaks when you cough or sneeze  Leaks when you are asleep  Leaks when you are physically active/exercising  Leaks when you have finished urinating and are dressed  Leaks for no obvious reason  Leaks all the time |
| **Variable:** | Question 1 of Wexner |
| **Definition:** | How often do you have accidents to solid, well-formed stool? |
| **Inclusion Criteria:** | *women scoring a 2 on INCONTSCREEN |
| **Type:** | Single Answer |
| **Response Options:** | 0 = Never  1 = Less than once per month  2 = Less than once/week & greater than once/month  3 = Less than once/day & greater than once/month  4 = Once a day or more than once a day |
| **Variable:** | Question 2 of Wexner |
| **Definition:** | How often do you have accidents to liquid stool/diarrhea? |
| **Type:** | Single Answer |
| **Response Options:** | 0 = Never  1 = Less than once per month  2 = Less than once/week & greater than once/month  3 = Less than once/day & greater than once/month  4 = Once a day or more than once a day |
| **Variable:** | Question 3 of Wexner |
| **Definition:** | How often does the gas escape without your knowledge or control? |
| **Type:** | Single Answer |
| **Response Options:** | 0 = Never  1 = Less than once per month  2 = Less than once/week & greater than once/month  3 = Less than once/day & greater than once/month  4 = Once a day or more than once a day |
| **Variable:** | Question 4 of Wexner |
| **Definition:** | How often do you wear a pad/depends or change underwear? |
| **Type:** | Single Answer |
| **Response Options:** | 0 = Never  1 = Less than once per month  2 = Less than once/week & greater than once/month  3 = Less than once/day & greater than once/month  4 = Once a day or more than once a day |
| **Variable:** | Question 5 of Wexner |
| **Definition:** | How much do the above answers alter your lifestyle or activities? |
| **Type:** | Single Answer |
| **Response Options:** | 0 = Never  1 = Less than once per month  2 = Less than once/week & greater than once/month  3 = Less than once/day & greater than once/month  4 = Once a day or more than once a day |
| **Variable:** | Pain with intercourse |
| **Definition:** | In the past 30 days, how much has pain affected your satisfaction with your sex life? |
| **Type:** | Single Answer |
| **Response Options:** | 0 = Have not had pain in the past 30 days  1 = Not at all  2 = A little bit  3 = Somewhat  4 = Quite a bit  5 = Very much |
| **Variable ID:** | Pre/Post Partum Depression (Mental Health) |
| **Variable:** | Question 1 of PHQ-2 |
| **Definition:** | Over the past 2 weeks, how often have you been bothered by any of the following problems?  Little interest or pleasure in doing things |
| **Type:** | Single Answer |
| **Response Options:** | 1 = Not at all  2 = Several days  3 = More than half the days  4 = Nearly every day |
| **Variable:** | Question 2 of PHQ-2 |
| **Definition:** | Feeling down, depressed or hopeless |
| **Type:** | Single Answer |
| **Response Options:** | 1 = Not at all  2 = Several days  3 = More than half the days  4 = Nearly every day |
| **Variable:** | Satisfaction with care |
| **Definition:** | How satisfied are you with the results of your care during your pregnancy?* |
| **Type:** | Single Answer |
| **Response Options:** | 0 = Very unsatisfied  1 = Unsatisfied  2 = Neither satisfied nor dissatisfied  3 = Satisfied  4 = Very satisfied |
