# Supplementary material for: Implementation of an international standardized set of outcome indicators in pregnancy and childbirth in Kenya: Utilizing mobile technology to collect patient-reported outcomes
Source: PLoS One. 2019 Oct 16;14(10):e0222978. doi: 10.1371/journal.pone.0222978 (PMC6795527; doi:10.1371/journal.pone.0222978)
Supplement: S4 File — Survey on patient reported outcomes completed by patient after delivery. (DOCX) [file pone.0222978.s004.docx]

**SI4. Survey #4.** Survey on patient reported outcomes completed by patient after delivery

| **Variable:** | Satisfaction with care |
| --- | --- |
| **Definition:** | How satisfied are you with the results of your care during during your labor and the birth of your baby? |
| **Type:** | Single Answer |
| **Response Options:** | 0 = Very unsatisfied  1 = Unsatisfied  2 = Neither satisfied nor dissatisfied  3 = Satisfied  4 = Very satisfied |
